# 

**Published:** 1817-05

**Authors:** 


### CORRESPONDENCE.

Prost's "Diseases, elucidated by Dissections," will shortly appear in the Original Department.

Dr. Denmark's interesting paper (with a drawing) on Phlegmatia Dolens in a male, is received, and will appear in our July number.

Various Articles for the "Porte-Feuille" are unavoidably postponed.

Mr. Morrison's Dissection of a Case of Measles is received; as is Medicina Laica.

*The Editors regret to observe, that the Plate, illustrative of Dr. Lee's Case of Lepra Arabum, is unavoidably deferred till their next Number, on account of the Artist not being able to complete it in time for this month's publication.*

☞ Authors and Publishers are requested to send Copies of new Works for Review as early as possible after their publication. They will be reviewed early in proportion.

